# Malignant Melanoma of the External Auditory Canal: A Rare Entity

**Published:** 2018-03

**Authors:** Yookarin Khonglah, Nabanita Das, Vandana Raphael, Ankit-Kumar Jitani, N.Brian Shunyu

**Affiliations:** 1 *Department of Pathology, NEIGRIHMS, Shillong, India.*; 2 *Department of Otorhinolaryngology, NEIGRIHMS, Shillong. India.*

**Keywords:** External auditory canal, HMB-45, Malignant, Melanoma

## Abstract

**Introduction:**

Although malignant melanomas (MM) are common in the head and neck region; primary malignant melanoma of the external auditory canal (EAC) is rare.

**Case Report:**

We present the case of a 50-year-old symptomatic man with a malignant melanoma of the external auditory canal, which clinically masqueraded as a haemangioma. The patient subsequently developed extensive loco-regional metastasis, requiring extensive surgery. We describe the clinical presentation, differential diagnosis, both clinical and pathological in terms of other pigmented lesions in the external auditory canal, detailed histopathology, and literature review.

**Conclusion:**

We want to emphasize the importance of immediate and adequate biopsy of any pigmented lesion in the head and neck region to rule out MM. Also, we emphasize the importance of deep biopsy for proper histopathological assessment in addition to distinguishing it from benign melanocytic nevi, in order to initiate treatment.

## Introduction

Malignant melanoma (MM) is a malignancy of pigment-producing cells or the melanocytes present predominantly in the skin. They are also found in the eyes, ears, GI tract, leptomeninges, and mucous membranes of the oral cavity and genital tract. The incidence of MM has continued to increase dramatically in the last few decades. This is due to increased exposure to sun in the general population. Head and neck melanomas are complex due to the anatomical complexity of this region and thus the treatment is also challenging. It is a lethal disease, as it accounts for 65% of all deaths from skin cancers (1). The majority of head and neck melanomas occur in the cheek (2) and only 7% of head and neck melanomas involve the ear (3). In the ear, the majority occur in the external ear (4). Involvement of the external auditory canal (EAC) is unusual and few cases are reported (5-7).

## Case Reports

**Fig 1:**
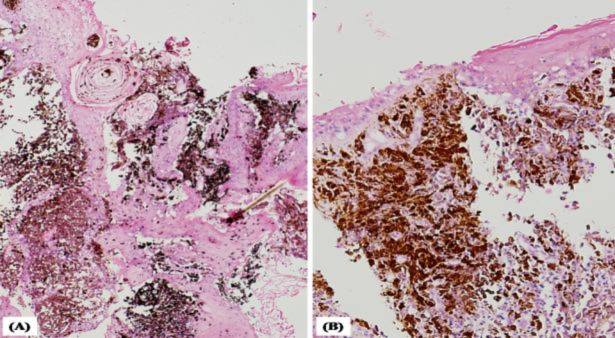
Punch biopsy from external auditory canal. (A) Strips of stratified squamous epithelium with underlying melanocytes in the external auditory canal (H&E, 40x); (B) higher magnification showing the melanocytes. (H&E, 200x)

**Fig 2:**
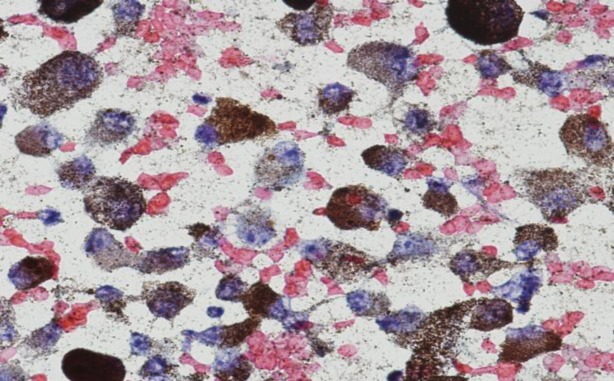
Fine needle aspiration of the parotid gland showing cells with prominent nucleoli and brown coloured melanin pigment (Pap stain, 400x)

An apparently healthy 50-year-old man presented to the ENT clinic with a 2-year history of blood stained discharge from his left ear. He also had pain and mild hearing loss. Examination revealed a bluish-black mass arising in the EAC. Examination of the right ear and the head and neck showed no other significant findings. There was no lymphadenopathy and no enlargement of the parotid gland. Appearance of the mass (bluish), presence of occasional bleeding and no other significant clinical findings led to the clinical diagnosis of haemangioma. Due to the clinical impression of a haemangioma, the mass was locally excised under local anaesthesia. Histopathological evaluation of this surgical specimen showed fragmented strips of stratified squamous epithelium with heavily pigmented melanocytic cells proliferating intraepidermally (Fig.1A). A small fragment of dermis also showed proliferation of melanocytic cells (Fig.1B). Melanin bleach confirmed that the pigment was melanin. Perls stain was negative, which ruled out haemosiderin. Based on these findings, a diagnosis of compound nevus was given. As the biopsy was superficial (local anaesthesia, anatomical location and route of surgery led to compromised accessibility and adequacy), the possibility of MM could not be excluded and the patient was recommended for a complete evaluation immediately. However, the patient failed to comply and was lost to follow up. Five months after the initial surgery, he presented with left sided parotid and preauricular lymph node swellings. Fine needle aspiration cytology of both the swellings showed heavily pigmented pleomorphic tumour cells with prominent nucleoli, suggesting metastatic melanoma to the parotid gland and lymph node (Fig.2). Examination of the rest of the body did not show evidence of any suspicious pigmented lesion. Subsequently, a complete surgical removal of the tumour was performed. Intraoperatively, the left EAC showed a pigmented mass involving a superficial part of the cartilaginous EAC with massive extension to the parotid gland. En bloc removal of the left EAC with the tumour and parotid gland with neck dissection was performed. A safe margin of 2 cm was left. 

**Fig 3:**
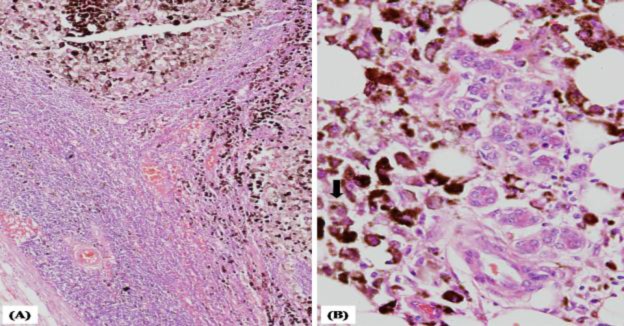
Excision specimen showing malignant melanocytes (A) within the lymph node; (B) intermixed with the parotid gland acini. (H&E, 200x)

The surgical specimen was sent for histopatho- logical examination. Microscopic examination of the mass in the left EAC showed an ulceroproliferative growth consisting of medium to large pigmented cells with prominent nucleoli. The tumour cells were seen to infiltrate the deeper subcutaneous tissue, parotid gland, and intra parotid lymph nodes (Fig.3A,3B). 

**Fig 4:**
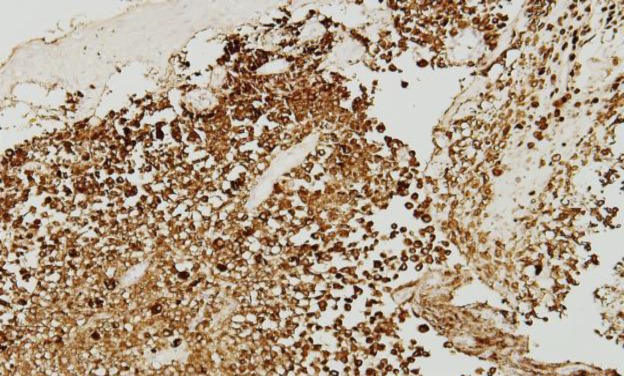
Immunohistochemistry with tumour cell showing positivity for HMB-45. (IHC, 100x)

The thickness of the tumour was 15mm (Clark level V). There was evidence of vascular and perineural invasion. Immunohistochemistry revealed that the tumour cells were positive for HMB-45 (Fig.4). The deeper cartilaginous part and bony margin of the EAC was free from tumour. Histopathological examination of the surgical specimen thus confirmed MM of the EAC with parotid gland and lymph node metastases. Surgery was followed by chemotherapy and radiotherapy.

## Results

MM is a lethal disease with a poor outcome. As MM was not a differential diagnosis in our case and the initial biopsy was superficial; a definitive diagnosis and therefore treatment, were delayed for about 5 months. Within that short time, our patient presented with extensive metastasis, which contributed to a poor outcome. This emphasises the importance of keeping MM in the differential diagnosis of pigmented cutaneous lesions at unusual sites.

## Discussion

Rare lesions in the EAC can range from benign to malignant tumours. Haemangiomas, benign vascular neoplasms, are commonly seen in the head and neck region. However, a literature search showed that haemangiomas are extremely rare in the EAC. They are commonly of two types: capillary and cavernous. In the EAC, cavernous haemangioma is common. Histopathological examination will show proliferation of large, tortuous vascular structures of varying sizes within abundant fibrous stroma. Surgical excision is the treatment of choice and long term follow up of reported cases did not show recurrence (8-10). Our patient was initially operated because of the misdiagnosis as haemangioma. Within 5 months, he suffered from recurrence and metastasis due to MM.

Nevus is a benign melanocytic lesion, which is the most common melanocytic lesion in the skin. However, it is extremely rare in the EAC. So far, only a few cases are reported in the literature. Nevus can also present with external ear symptomatology with a pigmented mass (11), which will closely mimic a MM. Thus, a full thickness biopsy is vital for the evaluation of suspicious lesions (1).

Malignant tumours are also rare in the EAC. Adenoid cystic carcinoma, squamous cell carcinoma, adenocarcinoma, basal cell carcinoma, mucoepidermoid carcinoma, etc… are seen in the EAC, but they are rare. Occurrence of MM in the EAC is even more rare than these tumours. Many published studies about malignant tumours of the EAC do not have a case of MM at this site (12,13). Jahn V et al. published a large series of ear melanomas. In that study, there were 161 cases; however, all occurred in the pinna (4). In pinna, the most common site was helix and antihelix. Ravin AG et al. had reviewed 199 patients with ear melanomas. They found that the most common type was superficial spreading (45.2%) and the most common site was the anterior helix (49.3%). Around 80% of their cases underwent wide local excision. Local recurrence rate with this treatment modality was found to be 10.6%. Overall incidence of local recurrence and metastatic spread was high (43.2%). Ulceration and increased thickness was associated with shorter survival. They concluded that surgical excision with confirmed negative margins (histopathologically) should be the aim for initial treatment (15,16). The initial treatment in our patient was thus inadequate. The subsequent surgical specimen showed an ulcerated tumour with a thickness of 15mm. These factors contributed to poor outcome in the patient.

The presenting feature of MM is non-specific, which does not contribute to raising suspicion unless a proper visual examination is performed. Patient may present with deafness, pain, bleeding, periauricular swelling, etc…. However, a mass is seen on visual inspection. In many reported cases, the initial diagnosis was of more common pathologies, such as aural polyp, ceruminous accumulation or infectious aetiology (5-7). In our case, a diagnosis of haemangioma was made as the patient had blood stained discharge with a mass.

Any cutaneous melanoma thicker than 4mm is considered to have a poor prognosis. Thickness of the lesion is the most sensitive indicator for development of metastasis and disease free survival. Ear MM is a high risk lesion as it has higher thickness at presentation (14). Poor prognosis of EAC MM may be due to the delayed diagnosis because of its hidden location, increased tendency for metastasis due to the thin epithelium and a short distance between the tumour and the draining lymphatics. The thickness of the tumour, in our case, 5 months after initial presentation, was found to be 15 mm and it was associated with parotid and lymph node metastasis. However, the initial removal was partial and biopsy was superficial.

The ABCDE criteria developed by The American Cancer Society are not useful as EAC is inaccessible for visual inspection by the patient. Irrespective of the site, the gold standard for melanoma diagnosis is histopathological examination of a full thickness biopsy. An excisional biopsy (or deep saucerisation technique) with narrow margins is preferred when possible. In an unsuspected case, if histopathological findings suggest a melanocytic nature of the tumour, the patient should be promptly taken for further work up and completion of resection; because surgery currently offers the best probability for cure of malignant melanomas of the head and neck region (1). Total surgical excision with at least 1-2 cm of safe margin is considered an optimal management. Considering the limited space and vital function of the EAC, the surgical resection may be challenging. The recurrence and mortality rates are still very high in this tumour. In our patient a safe margin of 2 cm was achieved and microscopic examination revealed that it was free from tumour. At 9 months, there is no evidence of recurrence and metastasis.

## Conclusion

Both benign and malignant tumours are rare in the EAC. MM is also rare and it has a poor prognosis with a high chance of recurrence and metastasis, so early diagnosis and prompt treatment is necessary. Hence, external ear symptoms with a pigmented mass should raise suspicion and a full thickness biopsy should be obtained for histopathological examination. 

## References

[1] Shashanka R, Smitha BR (2012). Head and neck melanoma. ISRN Surg.

[2] Bodenham DC, Chambers RD (1975). Malignant melanoma of the head and neck. Cancer of the head and neck.

[3] Byers RM, Smith JL, Russell N, Rosenberg V (1980). Malignant melanoma of the external ear Review of 102 cases. Am J Surg.

[4] Jahn V, Breuninger H, Garbe C, Moehrie M (2006). Melanoma of ear: prognostic factors and surgical strategies. Br J Dermatol.

[5] Gowthami C, Kumar P, Ravikumar A, Joseph L D, Rajendiran S (2014). Malignant melanoma of external auditory canal. J Clin Diagn Res.

[6] Hannan SA, Parikh A (2006). Malignant melanoma of the external ear canal. Lancet.

[7] Lin CH, Tu TY, Chu PY, Chen YW (2009). Malignant melanoma of the external auditory canal. Tzu Chi Med J.

[8] Feng HM, Chen HC, Shi ZP, Chang YM, Wang CW (2013). Cavernous Hemangioma of the External Auditory Canal: Two Case-Reports and a Literature Review. Int Adv Otol.

[9] Martines F, Bentivenga D, Maira E, Marasa S, Ferrara S (2012). Cavernous haemangioma of the external auditory canal: clinical case and review of the literature. Acta Otorhinolaryngol Ital.

[10] Verret DJ, Cochran SC, DeFatta RJ, Samy RN (2007). External Auditory Canal Hemangioma: Case Report. Skull Base.

[11] Katarkar A, Jain A, Shah P, Shah M (2011). A rare case of intradermal nevus of external auditory canal presenting with otorrhoea. Indian J Otol.

[12] Wang F, Wu N, Hou Z, Liu J, Shen W, Han W (2015). Diagnosis and treatment of rare malignant tumors in external auditory canal. Lin Chung Er Bi Yan Hou Tou Jing Wai Ke Za Zhi.

[13] Zhen S, Fu T, Qi J (2014). Diagnosis and treatment of carcinoma in external auditory canal. J Otol.

[14] Milbrath MM, Campbell BH, Madiedo G, Janjan NA (1998). Malignant Melanoma of the External Auditory Canal. Am J Clin Oncol.

[15] Langman AW, Yarington CT, Patterson SD (1996). Malignant melanoma of the external auditory canal. Otolaryngol Head Neck Surg.

[16] Ravin AG, Pickett N, Johnson JL, Fisher SR, Levin LS, Seigler HF (2006). Melanoma of the ear: treatment and survival probabilities based on 199 patients. Ann Plast Surg.

